# Drug-Induced Pneumonitis Secondary to Ribociclib in a Patient With Metastatic Breast Cancer

**DOI:** 10.7759/cureus.106954

**Published:** 2026-04-13

**Authors:** Zoha Mustafa, Maisoun Ahmad, Mohammed Alkhatib, Irfan Ul Haq, Mona Allangawi, Merlin Thomas, Mansoor Hameed

**Affiliations:** 1 Medicine, Royal College of Surgeons in Ireland, Manama, BHR; 2 Pulmonary Medicine, Hamad Medical Corporation, Doha, QAT; 3 Medicine, Qatar University, Doha, QAT; 4 Medicine, Weill Cornell Medicine - Qatar, Doha, QAT; 5 Pulmonary Medicine, Hamad General Hospital, Doha, QAT

**Keywords:** cdk4/6 inhibitors, interstitial lung disease, metastatic breast cancer, pneumonitis, ribociclib

## Abstract

Ribociclib, a CDK4/6 inhibitor, is vital in the management of hormone receptor-positive (HR+), human epidermal growth factor receptor 2-negative (HER2-) metastatic breast cancer. However, drug-induced pneumonitis is a rare but serious adverse effect that requires timely identification and intervention.

Our case presents a 66-year-old woman with stage IV metastatic breast cancer on ribociclib presenting with a two-month history of worsening dry cough and progressive dyspnea. Computed tomography of the chest revealed bilateral interstitial lung changes. Bronchoalveolar lavage ruled out infectious causes, and pulmonary function tests indicated a restrictive pattern, all supporting the diagnosis of drug-induced pneumonitis.

Discontinuation of ribociclib and initiation of corticosteroid therapy rapidly improved the patient’s symptoms, and by the time of her discharge, her oxygen saturation was stable.

This case emphasizes the importance of early recognition of pneumonitis in patients on CDK4/6 inhibitors. Clinicians should maintain vigilance for pulmonary symptoms and consider rapid intervention to prevent severe outcomes.

## Introduction

Ribociclib is a selective CDK4/6 inhibitor approved for the treatment of hormone receptor-positive (HR+)/human epidermal growth factor receptor 2-negative (HER2-) advanced and metastatic breast cancer. By inhibiting the phosphorylation of the retinoblastoma protein, ribociclib induces cell-cycle arrest at the G1-S checkpoint [1]. While its efficacy in prolonging progression-free survival is well-established, emerging evidence has identified interstitial lung disease (ILD) and pneumonitis as rare but serious adverse effects.

Recent meta-analyses and reports have demonstrated an increased risk of ILD associated with CDK4/6 inhibitors, including ribociclib [2,3]. Regulatory agencies have issued safety warnings highlighting the potential severity of these reactions. We describe a case of ribociclib-induced pneumonitis to further contribute to the growing body of literature and emphasize the importance of early diagnosis and management.

This report adheres to the CARE guidelines for case reports.

## Case presentation

A 66-year-old female with stage IV HR+/HER2- metastatic breast cancer with lung and bone involvement was on hormonal and targeted therapy, including ribociclib and letrozole. She had a history of morbid obesity, type 2 diabetes mellitus, and hypertension. She was taking oral hypoglycemic agents for her diabetes and managing her hypertension with diet changes. The patient had been receiving ribociclib at a dose of 600 mg once daily, administered in a 28-day treatment cycle consisting of 21 days on therapy followed by a 7-day break (21/28 schedule). Prior to the onset of symptoms, the patient had completed a total of 29 treatment cycles of ribociclib. She was a lifelong non-smoker and had no relevant environmental or occupational exposures.

She presented with a dry cough for two months that had progressively worsened over two weeks, accompanied by exertional dyspnoea. She denied fever, chest pain, hemoptysis, orthopnoea, weight loss, or night sweats. On examination, she was afebrile with a blood pressure of 150/90 mmHg, and oxygen saturation was 95% on room air at rest, decreasing to 88% after minimal exertion. Lung auscultation was unremarkable.

**Figure 1:**
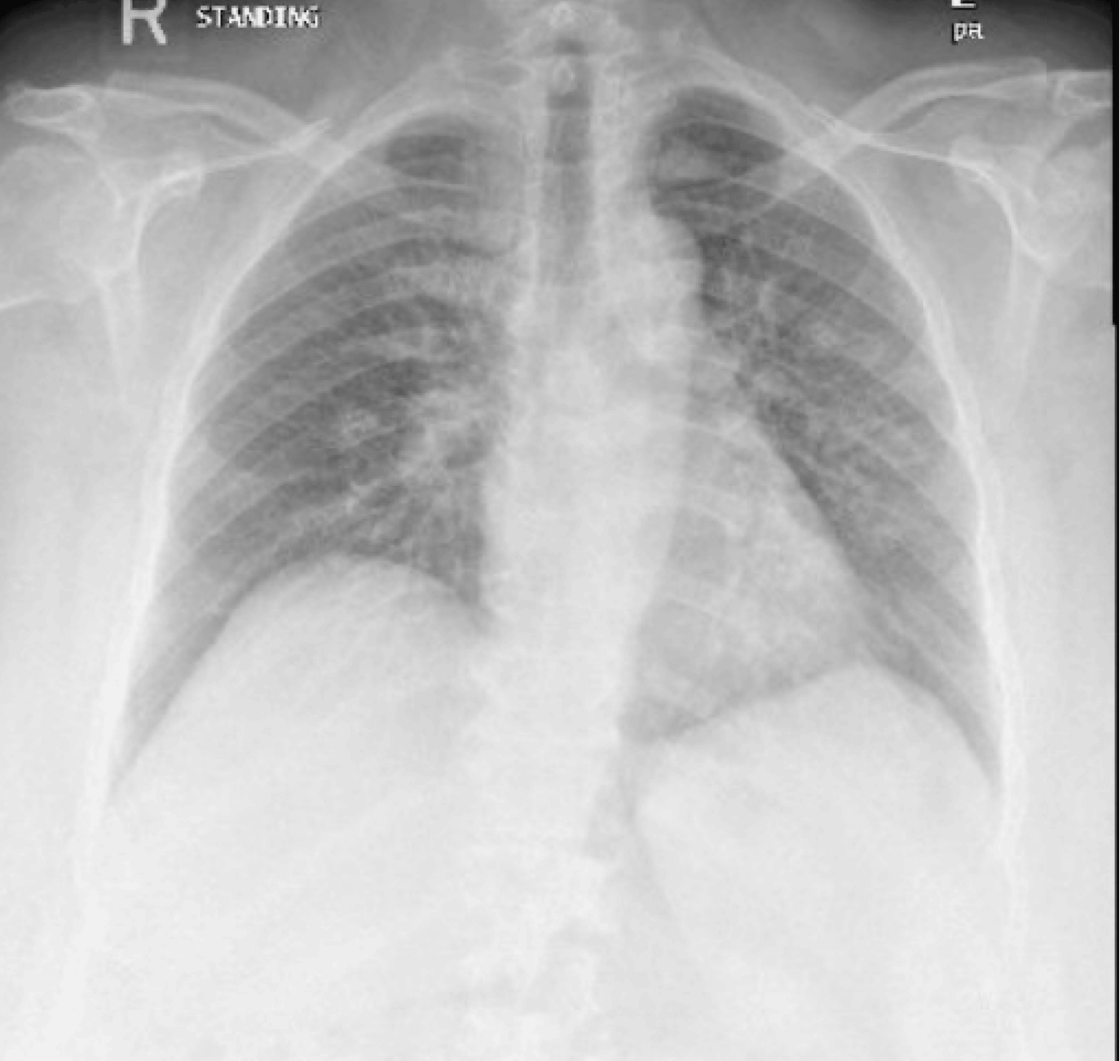
Chest X-ray showing grossly normal lung parenchyma with no obvious pulmonary pathology

**Figure 2:**
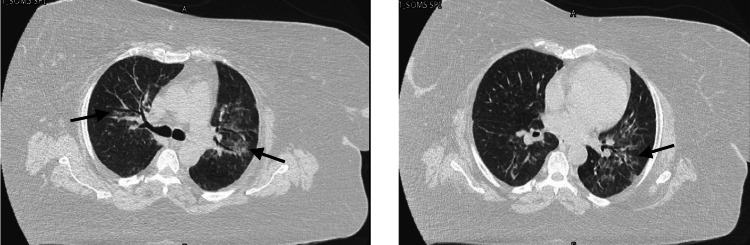
High-resolution CT of the chest demonstrates bilateral patchy ground-glass opacities predominantly involving the upper lobes (left panel), with more pronounced subpleural ground-glass opacification in the left lower lobe (right panel), as indicated by the arrows

Physical examination showed clear lung fields bilaterally, regular heart sounds with no murmurs, no lower limb edema, and a soft, non-tender abdomen. Chest X-ray revealed no abnormalities (Figure 1). Laboratory results showed leukopenia (WBC 3.1 × 10^9/L) and elevated CRP (81 mg/L), consistent with systemic inflammation. Autoimmune serology was unremarkable. High-resolution computed tomography of the chest demonstrated bilateral, upper lobe predominant, patchy areas of ground glass change with some subpleural as well as centrilobular opacification in keeping with inflammatory change (Figure 2). A comprehensive infectious workup, including viral polymerase chain reaction (PCR) and bronchoalveolar lavage (BAL), was performed, revealing a negative infectious workup for bacterial, fungal, and viral pathogens. BAL cytology demonstrated a differential cell count consisting of 90% macrophages, 7% lymphocytes, and 2% neutrophils. Pulmonary function testing showed a moderate restrictive ventilatory defect with reduced lung volumes (forced vital capacity (FVC) ~59% predicted, total lung capacity (TLC) ~50% predicted) and moderately reduced diffusing capacity (diffusion capacity of the lungs for carbon monoxide (DLCO) ~51% predicted).

Based on clinical, radiological, and laboratory findings, a diagnosis of ribociclib-induced pneumonitis was made. Causality assessment using the Naranjo Adverse Drug Reaction Probability Scale classified this event as “probable”, based on the temporal association, exclusion of alternative causes, improvement following drug withdrawal, and prior reports of ribociclib-associated pneumonitis [4]. Based on the Common Terminology Criteria for Adverse Events (CTCAE), the pneumonitis in this case was consistent with grade 2 toxicity, given the presence of symptomatic dyspnoea limiting exertion without the need for supplemental oxygen at rest [5].

**Figure 3:**
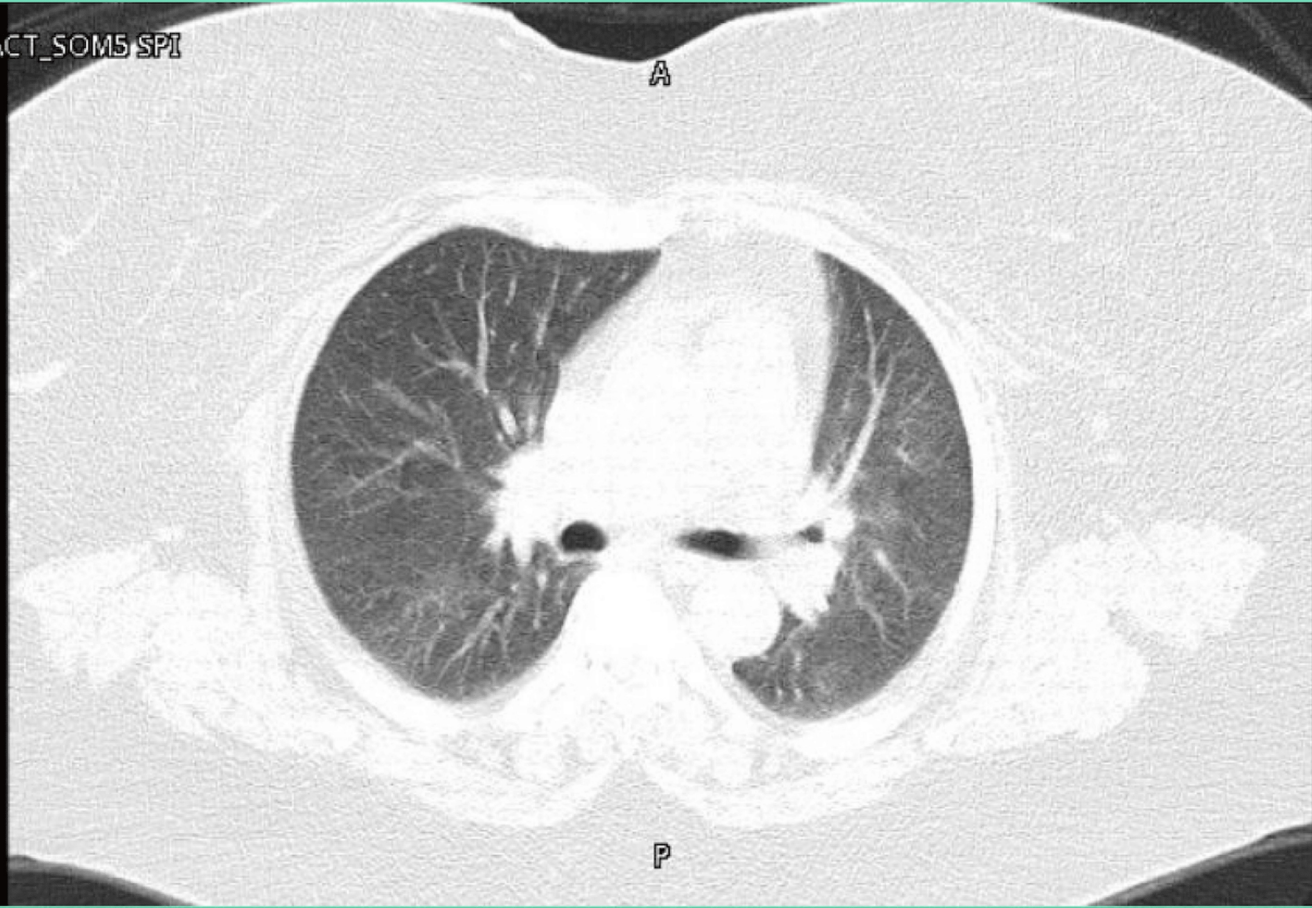
Follow-up chest CT at eight weeks showed complete resolution of the previously observed changes

Ribociclib was permanently discontinued, and the patient was treated with oral prednisolone 40 mg daily followed by a gradual taper, resulting in marked clinical improvement. She continued with letrozole alone. She was discharged with planned outpatient follow-up, including repeat chest imaging and pulmonary function tests (PFTs) to assess recovery and guide further management. Chest CT obtained after eight weeks revealed interval improvement in the previously noted radiological abnormalities (Figure 3). Repeat PFT performed two months later demonstrated interval improvement in lung volumes and gas transfer, with FVC increasing from 59% to 65% predicted, TLC from 50% to 58% predicted, and DLCO from 51% to 63% predicted. Ribociclib was not rechallenged. Subsequently, the patient was switched to palbociclib, which she has tolerated well without recurrence of respiratory symptoms to date.

## Discussion

Pathophysiology of drug-induced pneumonitis

Pneumonitis refers to inflammation of the lung tissue and can be infectious (caused by pathogens) or non-infectious (caused by drugs, allergens, or autoimmune conditions). This condition can cause fluid accumulation and lung parenchyma damage, leading to symptoms like cough, dyspnea, chest pain, and fatigue. Drug-induced pneumonitis is a type of interstitial lung disease (ILD), and the radiological and laboratory findings, including the Narango probability score, supported this diagnosis in our patient. Alternative diagnoses, such as infection, lymphangitic carcinomatosis, and cardiac failure, were excluded.

Over 450 suspected drugs can potentially cause ILD as a side effect [6]. The exact mechanisms for drug-induced ILD are not known, but proposed processes include direct cytotoxicity to alveolar epithelial or capillary endothelial cells or immune dysregulation. In the case of ribociclib, the drug may act as an antigen (or hapten), triggering an immune response with the activation of inflammatory cytokines and the infiltration of immune cells in the lung parenchyma [7]. This correlates with the radiographic images of our patient: the ground-glass opacities indicate alveolar filling with fluid or inflammatory cells [8].

In a study by Birnhuber et al. (2020) performed on mice with bleomycin-induced pulmonary fibrosis, CDK4/6 inhibitors were found to increase pulmonary inflammation through enhanced inflammatory cell recruitment [9]. It is also proposed that the inhibition of CDK4/6 causes cell senescence and a senescence-associated secretory phenotype (SASP), increasing inflammatory cytokines, growth factors, and proteins, leading to pulmonary toxicity [10].

Incidence and risk factors

Across clinical trials, pneumonitis or ILD was reported in about 1.6% of patients treated with ribociclib, with 0.4% developing grade 3-4 events. However, real-world data suggest a higher incidence of ~2-3%. Emerging evidence from recent studies has highlighted awareness of these potentially fatal side effects [11]. The FDA issued a warning in 2019 that CDK4/6 inhibitors, including ribociclib, may cause rare but severe lung inflammation [12]. Healthcare providers should monitor for symptoms like hypoxia, cough, or dyspnea to prevent complications. In a study by Ilhan et al., 90% of patients discontinued ribociclib due to ILD-related issues [13].

Real-world data suggest that the incidence of CDK4/6 inhibitor-associated ILD may be higher than reported in clinical trials. A Turkish multicenter study involving 464 patients reported an incidence of 2.1%, with hypersensitivity pneumonitis and nonspecific interstitial pneumonia (NSIP) as common patterns. Notably, no associations were found with age, smoking status, or the presence of lung metastases, suggesting that ethnic/regional factors may contribute to susceptibility [13]. In Asian cohorts, incidence reaches 1-3.3% with a median onset of approximately 75 days, suggesting genetic or environmental modifiers may influence risk [14]. In contrast, our Qatari patient demonstrated a delayed onset at 29 months of ribociclib exposure, representing one of the longest reported durations prior to onset, as most cases occur within the first 12 months of therapy. This represents a rare, late-onset presentation, potentially related to cumulative exposure or senescence-associated secretory phenotype (SASP) mechanisms [9,10].

Her metabolic comorbidities may be relevant, as it aligns with the FDA Adverse Event Reporting System (FAERS) pharmacovigilance data showing an intermediate ILD risk for ribociclib compared with abemaciclib (highest risk) and palbociclib (lowest risk), further supporting individualized risk assessment [15].

Management and comparison to the literature

Discontinuing ribociclib and initiating corticosteroids is the standard management, as adopted in our case and others (e.g., Algwaiz et al., where symptoms improved within one week [16]). Corticosteroids suppress inflammatory responses, reducing alveolar and interstitial inflammation. While effective in most cases, some patients do not respond and may experience fatal outcomes [17]. These fatal outcomes have been described in approximately 0.4-1% of cases, underscoring the potential severity of this adverse event [13,17]. Successful switching to palbociclib following ribociclib-induced ILD, as observed in our patient, has been reported in other cases without recurrence of pulmonary symptoms, supporting non-class-wide toxicity [16]. A Japanese case series further emphasizes the role of early high-resolution CT surveillance, particularly in patients considered at higher risk, to facilitate early detection and improve outcomes [14].

Table 1 compares our case with two recent reports, providing insight into ribociclib- induced ILD. All involved women with HR+/HER2- breast cancer had common symptoms of dry cough and dyspnea. All required discontinuation and corticosteroids. Rechallenging ribociclib carries risks, as seen in Rajendran et al., where symptoms recurred [18].

**Table 1:** Two cases of ribociclib-induced interstitial lung disease (ILD), as compared to our case HR+: hormone receptor-positive; HER2-: human epidermal growth factor receptor 2-negative

Study/Case	Age/Sex	Cancer Type	ILD Type	Infectious or Non-Infectious	Symptoms	Imaging Findings	PFT Findings	Duration on Ribociclib	Toxicity Grade (CTCAE)	Management	Outcome
Our case	66/F	HR+/HER2- metastatic breast cancer (lungs, bones)	Pneumonitis	Non-infectious	Progressive dry cough, dyspnea	Bilateral interstitial lung changes with ground-glass opacities on HRCT	Restrictive pattern	29 months	Grade 2	Ribociclib discontinuation, corticosteroids (prednisolone 40 mg, tapering)	Significant improvements in symptoms
Algwaiz et al. [16]	46/F	HR+/HER2- metastatic breast cancer	Pneumonitis	Non-infectious	Acute chest pain, dyspnea	Pulmonary embolism with bilateral ground-glass opacities on CT	Not reported	~4 weeks (initial)	Grade 3	Ribociclib discontinuation, corticosteroids (methylprednisolone 1.5 mg/kg/day, tapering)	Symptom resolution
Rajendran et al. [18]	63/F	HR+/HER2- metastatic breast cancer	Organizing pneumonia	Non-infectious	Dry cough, exertional dyspnea	Bilateral mid- and lower-zone non-homogeneous opacity; patchy subpleural airspace opacities on CT	Moderate restriction	~3 months (initial)	Grade 2	Ribociclib discontinuation, corticosteroids (prednisolone 50 mg/day, tapering), rechallenged but stopped again	Initial symptom resolution, but symptoms reappeared after rechallenging

Other literature on CDK4/6 inhibitors shows similar conclusions. Mathew et al. reported pneumonitis with palbociclib after three months, and Okayasu et al. with abemaciclib after five months [10,19]. A disproportionality analysis by Cheng et al. found pulmonary toxicity most associated with abemaciclib, followed by ribociclib, and least by palbociclib [20].

## Conclusions

This case highlights the importance of the early detection and prompt management of drug-induced pneumonitis in patients on targeted therapies like ribociclib. While ribociclib is an effective first-line treatment for metastatic breast cancer, it can have severe pulmonary side effects that may be fatal. Early intervention with discontinuation and corticosteroids leads to significant improvements and prevents irreversible lung injury. Collaboration between oncologists and pulmonologists is essential, and respiratory symptoms should be monitored carefully. Further research is required to understand the pathophysiology, identify high-risk groups (e.g., ages, races, menopausal status), and develop more effective treatment plans.
